# Lifetime congenital urologic care: centering patient voices

**DOI:** 10.1186/s41687-026-01141-x

**Published:** 2026-07-09

**Authors:** Xinyuan Zhang, Annaliese C. Ionson, Ashley C. Groth, Kimberly A. Allen, Riley Adams, Thomas Vincent, Liz Sage, Molly DeWitt-Foy, Daniel Wood, Judith C. Hagedorn, Hadley M. Wood, Lindsay A. Hampson

**Affiliations:** 1 Glickman Urological Institute, Cleveland Clinic, Cleveland, USA; 2 Department of Urology, University of Washington, Seattle, USA; 3 The Unsilenced Movement, Aurora, USA; 4 Dalla Lana School of Public Health, University of Toronto, Toronto, Canada; 5 Adult Patient Advisory Council, Association for the Bladder Exstrophy Community, Vero Beach, USA; 6 Department of Urology, University of Washington, Seattle, USA; 7 Department of Urology, University of Colorado School of Medicine, Aurora, USA; 8 Department of Urology, University of California, San Francisco, USA

**Keywords:** Patient-centered outcomes, Patient engagement, Research prioritization, Lifetime congenital urologic care, Transitional urology, Reconstructive urology, Bladder exstrophy, Vesicoureteral reflux, Medical PTSD, Qualitative analysis

## Abstract

**Background:**

Adults with congenital urologic conditions, such as bladder exstrophy and vesicoureteral reflux, often require lifelong care that begins with childhood surgical reconstructions and extends through adolescence and adulthood with ongoing surveillance, follow-up care, and revision procedures when needed. Measurement of “success” can be difficult to quantify, as the definition of success may differ between parents and patients later in adulthood, and the definition of success should extend beyond f technical success to the broader care interventions and long-term outcomes, which often requires decades of follow-up time. The traditional clinician-defined endpoints may not capture what patients consider as “success” in the context of the adult patients’ evolving goals for urinary function, sexual health, fertility, body image, and mental health across the lifetime. Patient-centered outcomes research offers a framework for centering patient voices, yet this has rarely been applied in adult congenital urology.

**Main body:**

Guided by the Patient-Centered Outcomes Research Institute (PCORI) foundational expectations in patient engagement, we developed a structured community-partnership methodology for the patient-centered research process in lifetime congenital urologic care. The framework includes six steps: (1) partnership formation; (2) inclusive recruitment and relationship building; (3) co-defining research questions; (4) collaborative data generation and interpretation; (5) dissemination and knowledge return; and (6) iterative reflection and process evaluation. We illustrate its application through two collaborations. First, with the Unsilenced Movement (UM), a patient advocacy group addressing medical trauma related to childhood voiding cystourethrogram testing, to qualitatively analyze patient narratives and co-interpret themes related to post-traumatic stress and healthcare avoidance; second, with the Association for the Bladder Exstrophy Community (ABEC) to co-design a patient-driven survey to assess goals, needs, and barriers across the lifespan for those living with bladder exstrophy. In both partnerships, patient representatives served as core research team members, co-authors, and co-presenters of findings, and ensured that patients’ lived experiences were translated accurately in a rigorous methodology.

**Conclusion:**

This framework shows how early and sustained collaboration with patient advocacy communities can reshape research prioritization, outcome selection, and interpretation of data in lifetime congenital urologic care. By centering patients’ lived experiences and defining success together, researchers can move beyond clinician-defined metrics towards outcomes that better reflect patient priorities. This methodology is adaptable to other congenital and chronic conditions requiring complex medical and urological care.

## Background

Adults with congenital urologic conditions such as bladder exstrophy, vesicoureteral reflux, and hypospadias represent a uniquely complex population. Their care begins in infancy or early childhood and extends across their lifetime, often involving multiple reconstructive surgeries and long-term follow-up [1]. Measurement of success after reconstruction, however, can be difficult to quantify. Not only are variables not clearly defined, but the required follow-up is often on the order of decades [2]. In addition, although an individual has a reconstruction that could be considered successful in childhood, they will likely have ongoing and evolving needs, goals, values, and changing perspectives on urinary function, sexual health, fertility and childbearing, body image, mental health, and social participation through adolescence and adulthood. Furthermore, conventional measures of success defined by physicians, such as renal function, patency rates and complication rates, may also not fully capture what patients value or how they judge the impact of their care.

Congenital urologic conditions also entail frequent and sometimes intense exposure to medical environments, including repeated genital examinations, invasive testing, hospitalizations and procedures. The cumulative weight of these encounters can have lasting psychological and behavioral effects, influencing trust in healthcare, engagement with the medical system and long-term health outcomes [3, 4]. 

Patient-centered outcomes research, as promoted by the Patient-Centered Outcomes Research Institute (PCORI), emphasizes partnership with patients and caregivers in setting research priorities, designing studies, and interpreting findings [5]. Although such approaches have been applied in many clinical areas, they are particularly critical yet underutilized in the adult congenital urologic population [6, 7]. To our knowledge, no prior framework has specifically been applied to centering the voices of adults with congenital urologic conditions across their lifespan. Much of the existing work in this space has appropriately focused on children, parents, and pediatric surgical outcomes. However, adults with congenital urologic conditions may define success differently from their pediatric providers, parents and caregivers, particularly as they assume increasing agency in their own healthcare and navigate priorities that evolve in adulthood. Out of necessity, many turn to or help create patient advocacy organizations to find information, community, and language for their unmet needs [8, 9]. These groups are natural partners for co-creating research that centers patients’ lived experiences in research prioritization and understanding patient-set measures of “success.” Our collaboration therefore emphasizes the adult patient voice as a distinct and essential perspective within lifetime congenital urologic care. Herein we describe a structured, community-partnership methodology for the patient-centered research process and illustrate its use through collaborations with two patient advocacy communities in adult congenital urologic care.

## The patient-centered research process

**Fig. 1:**
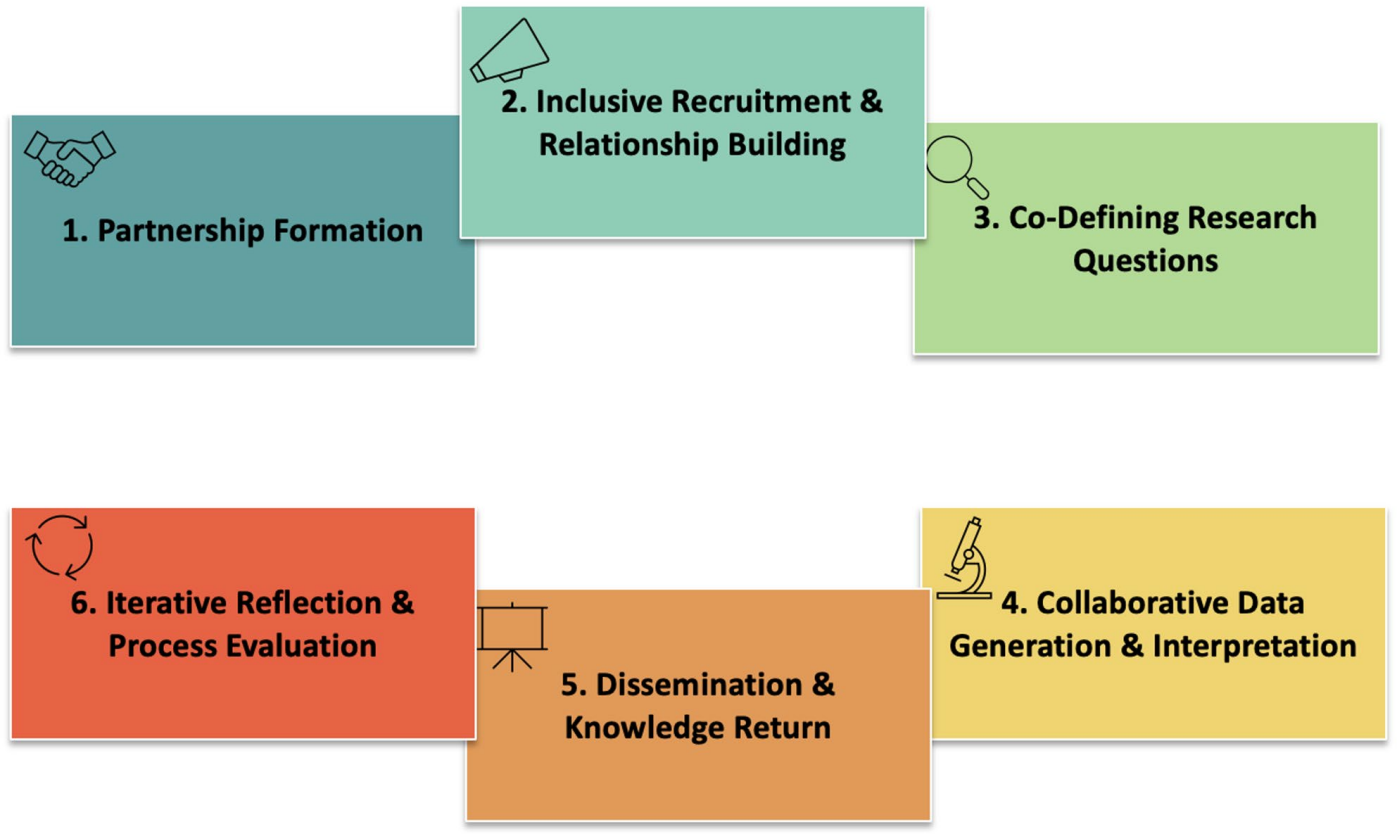
A structured framework for research that ensures meaningful and ongoing engagement of patients

This approach (Fig. 1) follows PCORI’s foundational expectations for partnership in research and positions patients and caregivers as partners throughout the research lifecycle, not as subjects of study [5]. We present a structured, pragmatic approach for applying the existing PCORI engagement principles to the lifelong congenital urologic care population.

The following collaborations are presented as applied examples of the patient-centered engagement process in the field of lifetime congenital urologic care:

1. **The Unsilenced Movement** (UM): a patient advocacy group founded in 2023 for patients and caregivers with medical trauma related to childhood voiding cystourethrogram (VCUG) testing. A collaboration was formed to perform qualitative thematic analysis of pre-collected patient narratives and extract themes that reflect the breadth and depth of the experienced trauma [8].
2. **Association for the Bladder Exstrophy Community** (ABEC): an international support network for patients and families living with bladder exstrophy. Focus group discussions were conducted to develop a survey to better understand patients’ goals and priorities across the lifespan [9].

### Partnership formation

**Fig. 2:**
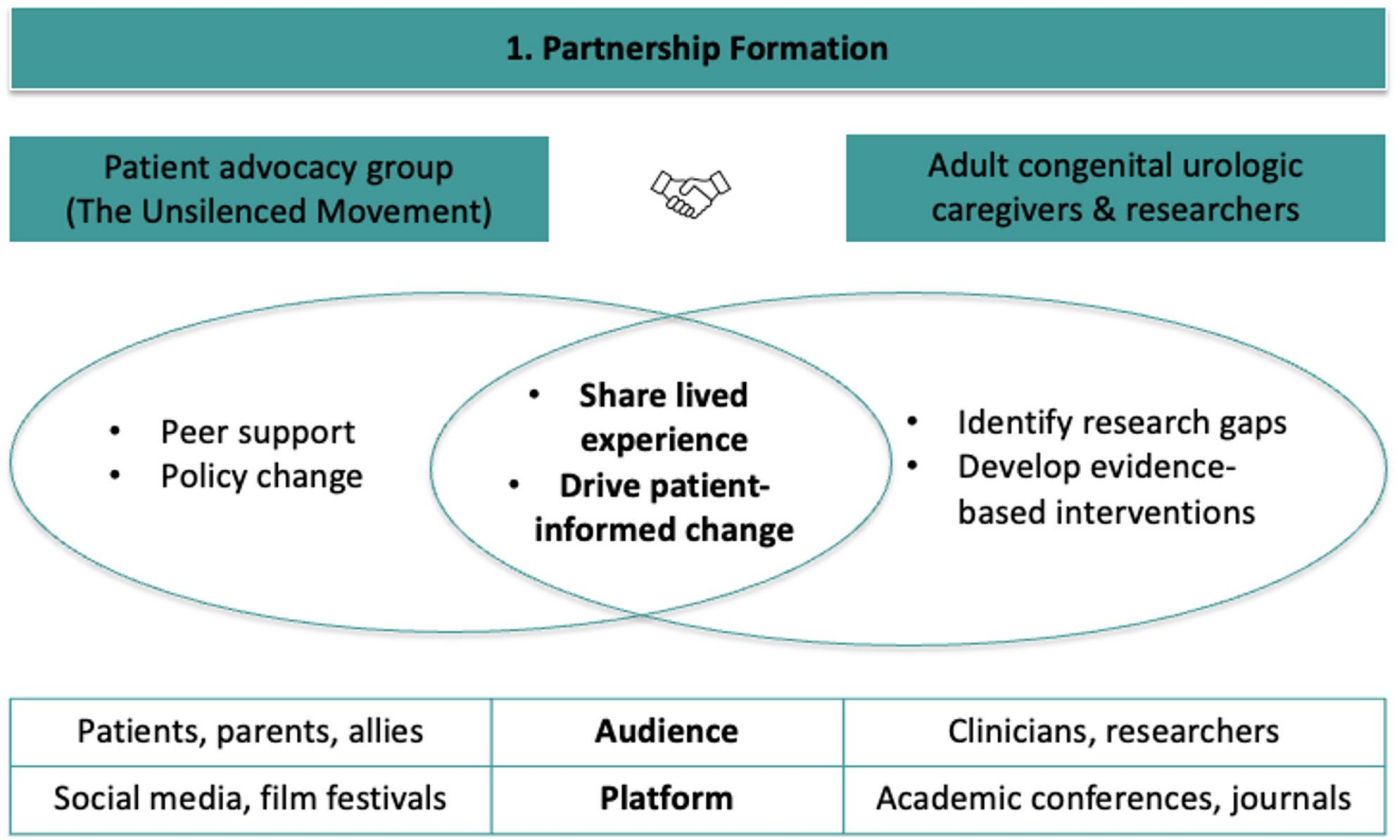
Illustration of each stakeholder’s individual goal, target audience, and platform, as well as establishment of shared goals during partnership formation with the UM group

Collaboration begins by identifying active patient organizations and stakeholders. Following PCORI’s foundational expectations for partnerships in research, diverse partners, including patients, advocates, clinicians, and social scientists, are engaged from an early stage [5]. The urologist-researcher group includes four academic urologists in the United States who have a clinical focus on caring for adults with congenital urologic conditions and recognize a gap in the literature in patient-centered outcomes and addressing the adult patients’ needs beyond traditional surgical outcomes. The research group recognized the importance of early and ongoing engagement of partners, another core PCORI principle for patient engagement, and reached out to potential community organizations prior to developing any specific research questions. By having an open dialogue with community partners to understand each stakeholder’s goals, all partners identified shared objectives and clarify values around trust, transparency, and accountability. In the collaboration with the UM group, UM members aimed to share patients’ firsthand lived experiences, provide peer support, and promote policy changes, whereas the adult congenital urologist-researchers sought to better understand the long-term impact of medical trauma and develop clinical tools to provide trauma-informed care for affected adults (Fig. 2). The ABEC community was created by parents of children with bladder exstrophy and sought better resources and support for a rare disease, and since its foundation, has a longstanding collaborative relationship on clinical care and research for children with bladder exstrophy. As more children with bladder exstrophy enter adulthood, the group has evolved to expand their continued support for adults with bladder exstrophy. Community partners and urologists had a shared desire to have longitudinal follow-up of patients and identify lifetime needs, barriers, and resources. Shared goals were established during the initial meetings of each partnership and revisited throughout the process to ensure all partners’ goals remained aligned.

We also found it important to establish a targeted audience, including what stakeholders felt were the most effective forms of information dissemination and platforms for each group. The UM group utilized storytelling, film, and social media to reach their targeted audience, with the goal of having published work that could be shared amongst its group members. Researchers focused on bringing rigorous qualitative methodology to this content, with the goal of presenting at academic conferences and publishing this work in peer-reviewed journals. Similarly, the ABEC partners wanted to develop and disseminate a patient-driven survey through their existing social network and generate content to return to the community.

### Inclusive recruitment and relationship building

**Fig. 3:**
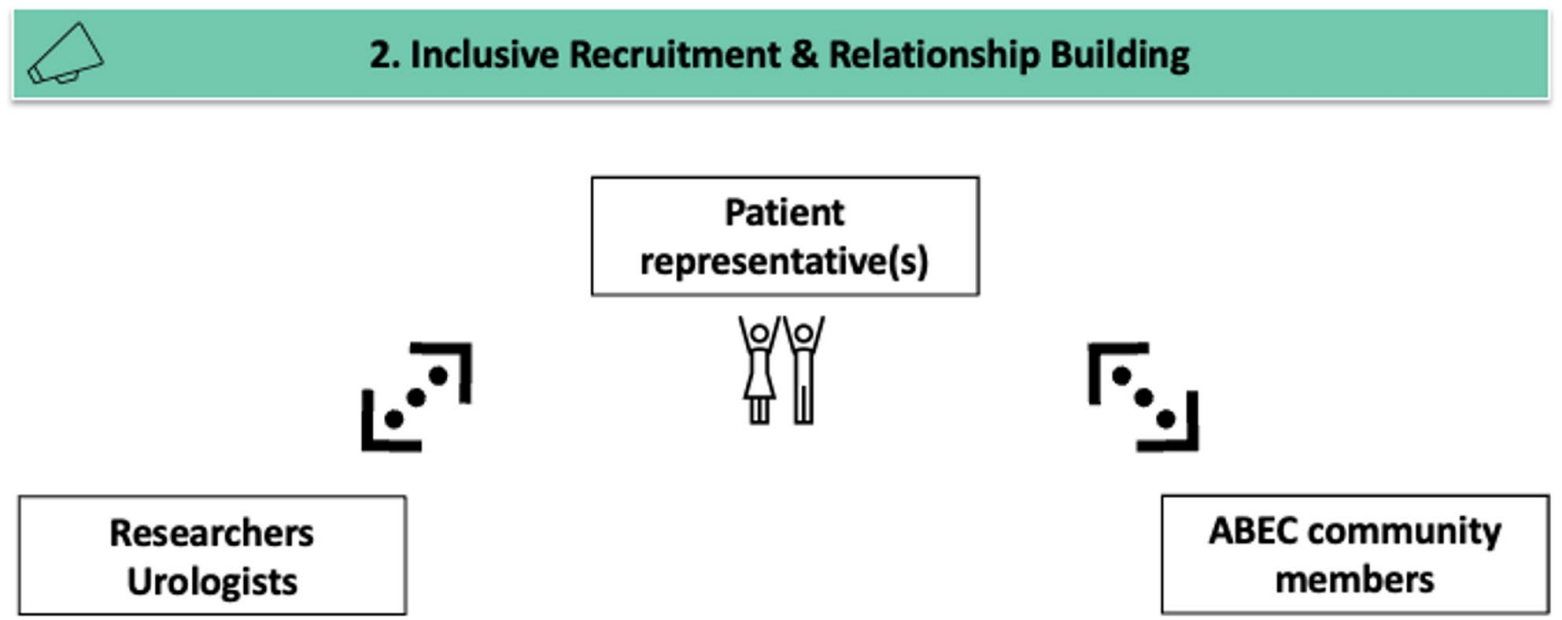
A figure illustrating relationship building through key patient representatives who serve as a connection between community members and researchers in the ABEC project

Outreach to stakeholder groups should aim to go beyond convenience sampling to include individuals whose experiences may have historically been marginalized or overlooked. Initial engagement with advocacy and support groups is inherently skewed towards those with the highest needs and most vocal opinions, as well as those with strong organizational structures. This can serve as a starting point to map out the landscape and identify salient themes and areas for future studies. Engagement of partners is framed as a mutual exchange, not one-sided data extraction. In the case of ABEC, there was a patient advocate and organization leader who served as the chief patient representative – a trusted member of the community with a vast patient outreach network (Fig. 3). The patient representative identified participants across age groups and geographic locations to participate in the study design. In both cases, patient representatives were core members of the research team, included in the development of study protocols, listed as official study personnel on all Institutional Review Board documents, and credited as authors in publications. The role of the patient representatives moved beyond advisory to active researchers and authors. Participants were provided ongoing updates and opportunities to shape subsequent phases via the patient representatives.

### Co-defining research questions

**Fig. 4:**
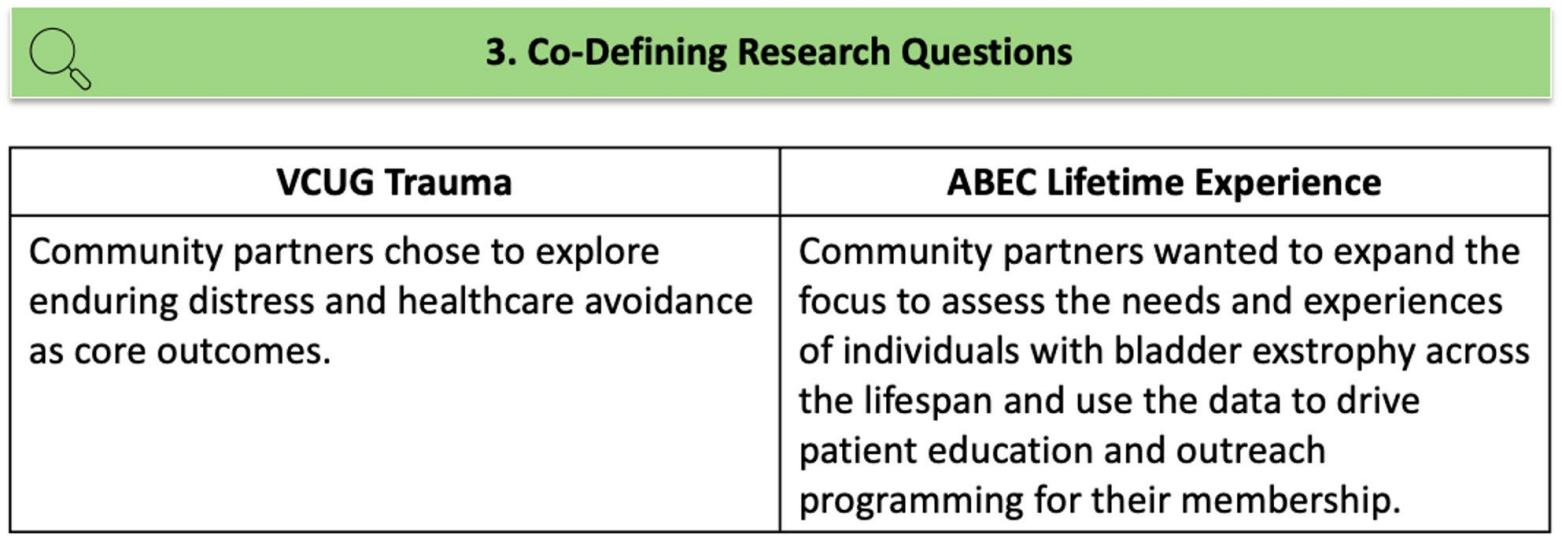
Examples of research priorities jointly set by community partners and academic researchers in understanding the impact of medical trauma associated with childhood VCUG testing, and goals for understanding the needs and experiences of adult bladder exstrophy patients across the lifespan, respectively

Research priorities emerge through structured dialogues and discussions, and community partners are helpful in identifying what questions and directions matter the most. Researchers, in partnership with stakeholders, can translate community priorities into methodologically sound questions without losing their original meaning. In the VCUG-related trauma study, partners prioritized post-traumatic stress disorder (PTSD) symptoms, long term psychological impact, and impact on future medical care as core outcomes, whereas community partners from ABEC expressed interest in expanding the focus to assess needs and experiences beyond childhood and throughout their lifespan (Fig. 4).

###  Collaborative data generation and interpretation

**Fig. 5:**
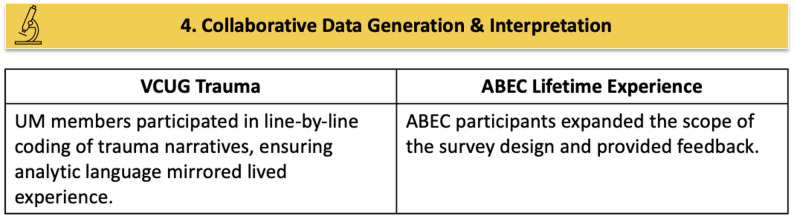
Examples of how community partners from the UM and ABEC groups participated in data generation, analysis, and interpretation

Qualitative and mixed-method approaches are useful, with community members actively involved in data collection, coding, and interpretation. If appropriate, patient partners can even serve as members of the research team to help guide interpretation to prevent misrepresentation. In both collaborations, patient narrative collection and focus group participation were initiated and led by the community partners, rather than from the academic researchers. Key patient representatives from each community partner distributed invitations clearly stating the purpose of collaboration, disclosed credentials of the research group, and obtained consent from community members to participate or have their narratives analyzed by the research team. In collaboration with UM, 44 patient narratives were collected via social media and published on the online platform regarding medical traumatic stress related to childhood VCUG testing [8]. A community member with a social science background was able to participate as a member of the coding team, engaging in line-by-line qualitative data coding of patient narratives, alongside the researchers, and all disputes were then resolved through group discussion or by the senior author. An additional member of the UM was able to participate in helping to shape the themes that emerged after coding was completed (Fig. 5). The results illustrated not only the spectrum and severity of negative impacts on psychological and physical health, but also highlighted successful and unsuccessful coping mechanisms, and gaps in current medical care settings in providing trauma-informed care. Similarly, in the collaborative work with ABEC, key patient representatives in the adult patient advisory council members reached out via the existing ABEC network to identify volunteers to participate in focus group discussion and reviewed drafts of questionnaires formulated by clinicians to identify areas in need of clarity or expansion. This included the addition of questions pertaining to mental health, distinguishing social from physical challenges in living with exstrophy, and identifying resource gaps. The review process was iterative and included both patients and parent-caregivers.

### Dissemination and knowledge return

**Fig. 6:**
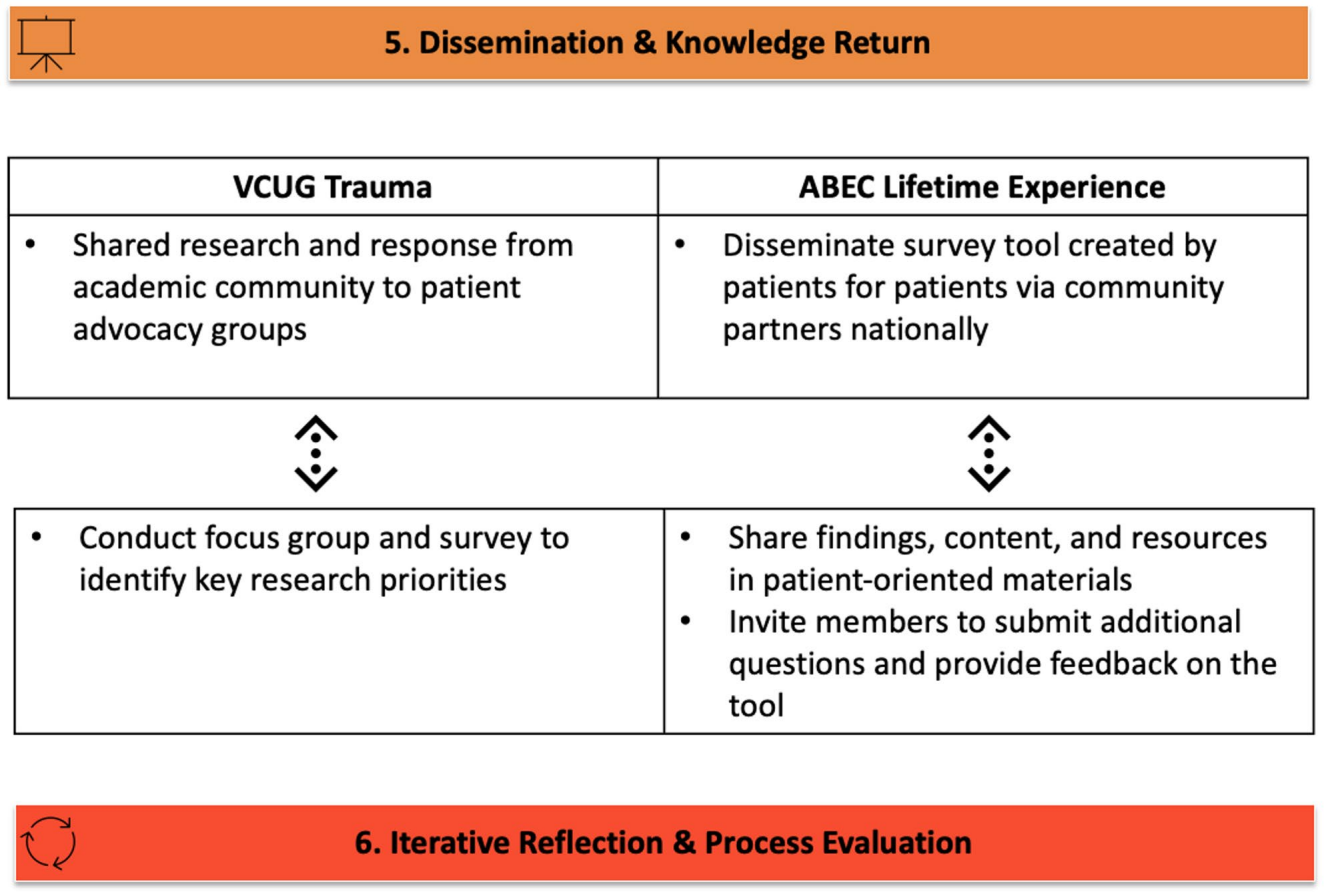
Illustration of the dissemination of knowledge and iterative reflection process in both collaborations with the UM and ABEC groups

It is important to define the form of dissemination that is important to each participant at the beginning of the research partnership. When possible, findings should be co-authored, co-presented, and co-disseminated. The use of multiple platforms such as academic conferences and journals, community forums, and advocacy networks is helpful to ensure that each partner in the research process reaches their desired audience, in the language and format best suited for that audience. When information is provided to research partners, there is often a flow of information back that helps to inform interpretation of research findings or direct future work. A key goal of the ABEC research group and patient participants is improving access to and transparency of survey responses for the larger community (Fig. 6). The ABEC research team will generate patient-driven reports of the survey responses and invite community members to submit additional research questions, share their analyses of the data, and provide feedback on the survey tool itself to promote continuous improvement. Data sharing supports a sense of community ownership and fuels future engagement.

### Iterative reflection and process evaluation

We applied another core PCORI principle in patient engagement in research to our research process by establishing an iterative process in collaboration. Continuous assessment ensures that engagement remains meaningful and sustainable. In both collaborations, researchers and community partners set up regular follow-up meetings were set up to evaluate the engagement process and gather feedback. Goals were reassessed and re-evaluated at these intervals to ensure continued alignment of partners and foster mutual learning across the team. Involving patients in identifying problems that are important to study is the first step in the research process, and despite various evidence-based principles and strategies to promote effective partnership in all stages of research, continued engagement of patients is often lacking [10]. Therefore, it is particularly important to include this last step in order to take the results and learnings from one iteration of the process, feed back to the community, and ensure continued, equitable and sustainable involvement throughout cycles of research.

## Outcomes & impact

The PCORI Engagement Rubric defines principles of engagement including reciprocity, co-learning, transparency, and trust, and analysis of PCORI-funded projects have shown that engagement of various stakeholders beyond clinicians improved research feasibility, acceptability, rigor, and relevance [11–13]. Within urology, transitional and lifetime congenital care remains one of the top research priorities within the field of urology; yet, there remains a large gap in current disease specific care model, quantitative assessment of care, and stakeholder perspectives [14]. By operationalizing the PCORI framework in two specific examples of disease and patient organizations in lifetime congenital urology research, we aim to illustrate a pragmatic framework to identify impactful patient-defined research priorities. Community-engaged research in this area can help define research questions, reshape what outcomes should be measured and how, and provide perspective on long-term urologic care for adults. By centering what patients identify as meaningful, research can move beyond traditional clinician-defined endpoints to reflect patient-defined, patient-reported outcomes. This strengthens trust between patient communities and academic medicine through shared ownership. Finally, the framework outlined here can be broadly applied to other patient populations within lifetime congenital urology.

## Lessons and challenges

This approach requires ongoing engagement from patients and advocates who are willing to partner with researchers and physicians. By partnering with patient advocacy organizations and identifying patient representatives as trusted mediators, more marginalized communities may be more likely to engage and partner with researchers. Capacity building, which involves identifying skills, strengths, and barriers to patient engagement, and providing training and support for both community partners and academic researchers, is essential for effective collaboration. Building this foundation takes time and resources, but it ensures the partnership is equitable and all voices can contribute meaningfully. Through the first iteration of the collaborative research with ABEC, the Lifelong Insights for Exstrophy Outcomes Survey was developed, publicized, and disseminated through our patient representatives to the entire ABEC patient community [15]. The results of this iteration was fed back to the community and we applied the same process to produce an easily accessible and searchable Adult Provider Directory for patients to find the correct provider after transitioning to adulthood [16]. Through the first round of collaboration with the UM group, qualitative analysis of patient narratives was performed, and top three categories, including medical traumatic stress, long-term psychological impact and barriers to medical care, were collectively chosen as the priority items to be published and shared with the academic community. Through this process, new research gaps were identified in developing tools to support individuals with medical traumatic stress in the adult care settings, and measures to help improve their care engagement. Capacity building with engagement of psychologists were identified as well, and would serve as the priority goal for the second iteration of this collaborative process.

At times, the goals of patient advocates and researchers may diverge, which reflects differences in priorities, motivations, and definitions of success. Early and ongoing dialogue helps to find mutual goals while respecting each partner’s unique perspectives. For instance, in collaborating with the UM group, many community member narratives expressed anger and distrust towards the medical system including urologists, advocated for banning VCUG as a test in the pediatric setting, and called for development of new tools to replace VCUG, whereas the research group focused on improving care to patients with existing trauma and sought collaboration with pediatric urologic providers. The community partner and research group openly discussed the importance of identifying areas of alignment and disagreement, established common ground on acknowledging the existing trauma in this population, maintained transparency about methodological constraints, and opened discussion when research decisions might affect the relevance or acceptability of the work. Initiatives by the UM group in technological development and policy reform, and research efforts on health system improvement in care transition across the lifespan were not included in this collaboration process. However, divergence in goals should not be viewed as a failure of collaboration, as these differences can help refine the research question and ensure final research outcomes remain both scientifically rigorous and meaningful to the community.

Patient advocacy organizations often provide space for the most impacted and vocal within the patient population. While their voices provide a crucial lens into understanding the lived experiences, they may not represent the entire spectrum within the community. Hence, it is important to seek input from affected patients who may exist outside the lead patient groups. Nonetheless, partnering with patient advocacy groups helps urologists and researchers understand the context of patient experiences, uncover needs, barriers, and perspectives, and inform researchers about the nuances of patient trust and community priorities.

Another consideration is that patients’ recollections of past experiences may not always align with the objective clinical events. However, we recognize that these memories and the associated meaning and impacts are central to the patient-centered approach. Whether or not the recollections of past experiences are completely factually accurate, they are the lived experiences of those individuals, and this impact is important to capture.

Finally, while tools for qualitative analysis are rapidly evolving, they remain imperfect in handling large volumes of narrative data and synthesizing insights with nuance and accuracy. Continued methodological innovation is needed to ensure complex patient narratives are translated into digestible and actionable findings without oversimplification.

## Future directions

Our next step is to use the insights learned from this process to propose larger-scale case-control designs in future studies to minimize inherent selection bias in the current approach. In addition, all caregiver stakeholders, including pediatric urologists, should be involved in the process. The emphasis of this work is to understand adults with medical trauma from childhood experiences and the evolving needs of adults who have undergone reconstructive surgeries in childhood is by no means a critique of pediatric urology practice. Rather, it reflects a feed-forward mindset to learn from patients’ lived experiences and improve adult and geriatric care. As patient goals, identities, and needs evolve over time, this approach emphasizes continuity, empathy, and long-term partnership between pediatric and adult providers to promote trauma-informed care.

## Conclusion

A patient-centered approach with intentional partnership, shared learning, and ongoing dialogue helps transform both the research content and process. By engaging patient groups as equal partners, we move beyond the traditional paradigm of observation toward collaboration that shapes priorities, methods, and outcomes in lifetime congenital urologic care.

## Data Availability

No datasets were generated or analyzed during the current study.

## Abbreviations

PCORI: Patient-Centered Outcomes Research Institute
UM: The Unsilenced Movement
VCUG: Voiding cystourethrogram
ABEC: Association for the Bladder Exstrophy Community
PTSD: Post-traumatic stress disorder
